# An Online MFL Sensing Method for Steel Pipe Based on the Magnetic Guiding Effect

**DOI:** 10.3390/s17122911

**Published:** 2017-12-15

**Authors:** Jianbo Wu, Hui Fang, Xiaoming Huang, Hui Xia, Yihua Kang, Chaoqing Tang

**Affiliations:** 1 School of Manufacturing Science and Engineering, Sichuan University, Chengdu 610065, China; wujianbo@scu.edu.cn (J.W.); huangxm@stu.scu.edu.cn (X.H.); xiahui@stu.scu.edu.cn (H.X.); 2 School of Mechanical Science and Engineering, Huazhong University of Science and Technology, Wuhan 430074, China; yihuakang@hust.edu.cn; 3 School of Electrical and Electronic Engineering, Newcastle University, Newcastle upon Tyne NE1 7RU, UK; c.tang2@newcastle.ac.uk

**Keywords:** steel pipe, online MFL (magnetic flux leakage) testing, non-ferromagnetic and ferromagnetic supports, magnetic guiding effect

## Abstract

In order to improve the sensitivity of online magnetic flux leakage (MFL) testing for steel pipe, a sensing method based on the magnetic guiding effect is proposed and investigated in this paper. Compared to the conventional contact sensing method using a non-ferromagnetic support, the proposed method creatively utilizes a ferromagnetic one to guide more magnetic flux to leak out. Based on Hopkinson’s law, the principle of the magnetic guiding effect of the ferromagnetic support is theoretically illustrated. Then, numerical simulations are conducted to investigate the MFL changes influenced by the ferromagnetic support. Finally, the probe based on the proposed method is designed and developed, and online MFL experiments are performed to validate the feasibility of the proposed method. Online tests show that the proposed sensing method can greatly improve the MFL sensitivity.

## 1. Introduction

As an important pressure component, steel pipes are widely used for oil and gas storage and transportation. With the development of the oil and gas industry, high-quality steel pipes are needed more and more. According to the American Petroleum Institute (API) standard, steel pipes need to be tested by nondestructive testing methods (NDT) [1]. Due to the development of high-speed continuous-rolling pipe mills, online NDT technology with high speed and high sensitivity for steel pipe is increasingly in demand. There are several NDT methods for steel pipes, such as ultrasonic testing (UT) [2,3], magnetic particle testing (MPT) [4], eddy current testing (ECT) [4,5,6], magnetic flux leakage testing (MFL) [4,7,8,9,10,11,12,13,14], and guided wave testing [15]. Among them, as a non-contact NDT (nondestructive testing) technology [16,17,18,19], MFL technology is a powerful and highly efficient method that has been widely used for ferromagnetic objects, such as oil-gas pipeline [7,8,9,10,11,12,13,14], rail track [20,21], steel wire [22,23,24], oil storage tank bottom [25,26] and bridge cable [27]. Besides this, MFL is not affected by the presence of non-ferromagnetic media around the specimen, so MFL inspection can still be performed when the surface of the specimen is not clean, such as when the surface has dirt or dust on it [11]. Due to these features, MFL has obvious advantages, particularly for the online inspection of steel pipes.

On the one hand, due to the development of high-speed continuous-rolling pipe mills, high-speed MFL testing for steel pipes is increasingly in demand. Some MFL technologies were developed to improve the testing speed. For steel pipes, the commonly used MFL techniques mainly include axial magnetization for circumferential defects and circumferential magnetization for longitudinal ones by relative helical scanning [28,29,30]. Then, aiming to improve the testing speed, new MFL technologies based on orthogonal magnetization [31] and unidirectional axial magnetization [32] were proposed to simplify the scanning motion. Further, the eddy current effect in high-speed MFL testing was investigated and some compensation methods were proposed [33,34,35,36,37,38,39].

On the other hand, improving the MFL sensitivity for steel pipes is another key issue. MFL is conducted on the basis of the physical phenomenon of a ferromagnetic specimen that, in a certain magnetized state, will produce magnetic flux leakage if any discontinuities are present in it. Then, a magnetic sensor is used to capture this leakage in the vicinity of the discontinuity. In order to realize high-sensitivity MFL testing, the magnetization method, sensor arrangement, and leaked magnetic flux enhancement are three key factors, which are as follows: (1) Magnetization method: There are three types of MFL methods, namely, DC MFL, AC MFL, and pulsed MFL [40,41,42]. DC MFL uses large direct currents to generate a static magnetizing field, thus, DC MFL based on direct currents is suitable for hard-magnetization objects, such as steel pipes. By applying an alternative current, AC MFL is generally sensitive to surface defects due to the skin effect, depending on the excitation frequency chosen, which is suitable for the detection of surface defects, such as surface cracks in steel bars. With pulsed MFL, the probe is driven with a pulsed current and the rich frequency components can provide information from different depths due to the skin effects, which is suitable for precise inspection with a slow testing speed; (2) Sensor arrangement: After the magnetic flux leaks out from the defect, a proper magneto sensor, such as a hall sensor [33,43], induction coil [44,45,46], giant-magnetoresistance sensor (GMR) [47,48], magneto fluid [49], etc. is chosen to pick up the perturbed magnetic field. Due to the lift-off effect, the leakage field rapidly decreases with increasing radial distance, hence, in order to realize high-sensitivity sensing, magnetic sensors are placed as closely as possible to the objects to be tested; (3) Leaked magnetic flux enhancement: A concentrating device made of ferromagnetic material is applied to affect the distribution of the magnetic flux leakage and concentrate more leaked magnetic flux into the sensor location, which can improve the sensitivity. Due to the bad surface condition of used drill pipes caused by attachments such as mud, rock, and oil, the conventional contact detection will evidently cause severe wear and even damage to the probe [43,45]. Hence, Ma and et al. proposed a method to improve the SNR with a magnetic concentrating device [43]. Wu and et al. proposed a lift-off-tolerant MFL sensor based on the magnetic field focusing effect of ferrite cores [45]. The aim of the above two investigations was to realize MFL inspection for drill pipes at distance, which is not suitable for steel pipes in the production line.

In online MFL testing for steel pipes, the DC magnetization method is usually used to produce a strong and uniform magnetizing field, which is suitable for both surface and sub-surface defect inspection at high speed. Different from the used drill pipe inspection with a bad surface condition, the steel pipe in the product line has a smooth surface, hence, the probe can be placed as closely as possible to the pipe surface to improve the sensitivity. A conventional method is as follows: First, the magnetic sensors are installed in a non-ferromagnetic protecting support with a certain thickness. Then, the non-ferromagnetic support is forced to contact the pipe surface directly. It can be seen that even for the contact method, there is still an inevitable lift-off distance from the pipe surface to the sensor location because of the thickness of the non-ferromagnetic support, restricting the sensitivity improvement, which is the main challenge of the conventional method. In this paper, a ferromagnetic support is creatively utilized to replace the conventional non-ferromagnetic one. When the non-ferromagnetic support is replaced by a high-permeability ferromagnetic support, the magnetic reluctance of leaking path from the defect to the sensor location is reduced greatly, and thereafter more leaked flux leakage is guided to the sensor location, leading to a higher sensitivity. Besides, the proposed sensing method is simple and achievable, and changes nothing to the conventional probe structure except the support material.

In this paper, based on Hopkinson’s law, the principle of the proposed method is theoretically illustrated in Section 2. Then numerical simulations are conducted to investigate the MFL changes influenced by the ferromagnetic support in Section 3. The probe based on the proposed method is designed and developed, and online MFL experiments are performed to validate the feasibility of the proposed method in Section 4. Finally, Section 5 gives the conclusions.

## 2. The Principle of the MFL Sensing Method Based on the Magnetic Guiding Effect

**Figure 1:**
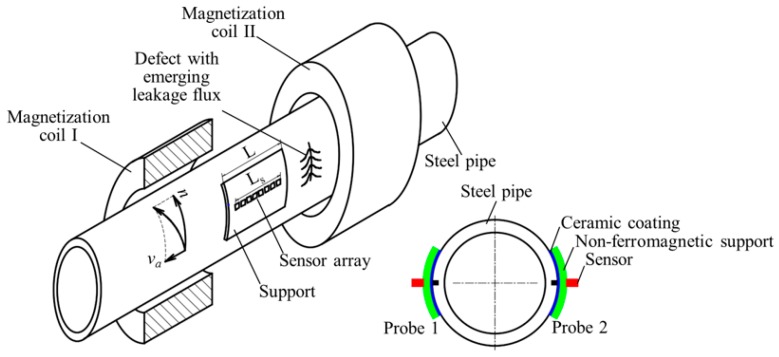
The magnetic flux leakage (MFL) inspection apparatus for steel pipe.

The principle of the MFL testing for steel pipes in a production line is schematically illustrated in Figure 1, which shows the testing apparatus and the pipe to be tested. To fulfill the 100% scanning coverage for circumferential defects, the pipe is passed through the testing apparatus in a helical motion. The steel pipe rotates with a rotational speed *n* and simultaneously conveys forward at a linear speed *v_a_*. A direct current flows through Helmholtz coils I and II and generates a magnetic voltage, which drives a magnetic flux through the pipe wall. If there are any circumferential defects in the pipe wall, leakage flux will be generated and detected by Probes 1 and 2, which are usually arranged symmetrically. In each probe, a sensor array with an effective length L_s_ is installed in a protecting support with a length L. Normally, the protecting support is made of non-ferromagnetic material, such as stainless steel. To avoid the sensitivity vibration caused by the pipe swing movement during the transportation line, the support needs to follow up the pipe’s movement and maintain a constant lift-off distance from the pipe surface. The support, with its internal diameter equal to the pipe external diameter, is usually forced to contact the pipe surface firmly by the external force supplied by a pressure string or cylinder, resulting in direct friction between the support and the steel pipe. To extend the service life of the probe, the contacting surface of the support is usually sprayed a high-hardness ceramic coating. Hence, there is still an inevitable lift-off distance from the pipe surface to the sensor location because of the thickness of the non-ferromagnetic support, restricting the sensitivity improvement, which is the main challenge of the conventional method.

**Figure 2:**
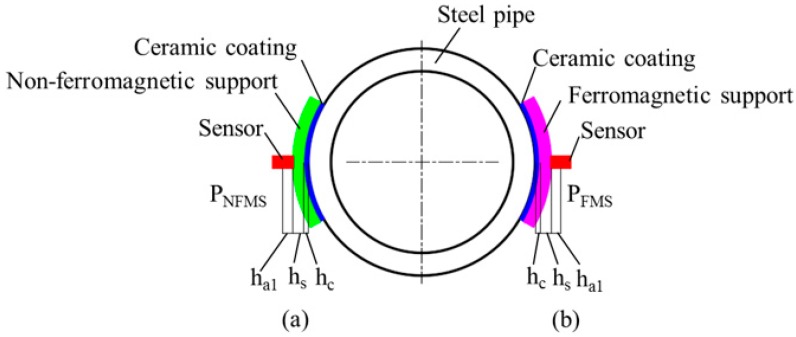
The schematic of MFL probe for steel pipe. (**a**) The probe with the non-ferromagnetic support (NFMS); (**b**) The probe with the ferromagnetic support (FMS).

The conventional contact method is schematically illustrated in Figure 2a. The magnetic sensor is installed in a non-ferromagnetic support (NFMS) with a thickness of *h*_s_. Due to the volume of the sensor, there is a distance *h*_a1_ from the support surface to the sensor measuring point. To extend the service life of the probe, the contacting surface of the support is usually sprayed a high-hardness ceramic coating with a thickness of *h*_c_. Finally, an inevitable lift-off distance *h*_0_ is formed, which is the sum of *h*_s_, *h*_a1_, and *h*_c_. Due to the lift-off effect, the inevitable lift-off distance *h*_0_ from the measuring point to the pipe surface will cause a low sensitivity, which is the main challenge of the conventional method. To solve this problem, in this paper a ferromagnetic support (FMS) is creatively applied to replace the NFMS, as schematically depicted in Figure 2b. When the NFMS is replaced by a ferromagnetic material, it will absolutely affect the MFL distribution and further change the sensitivity.

**Figure 3:**
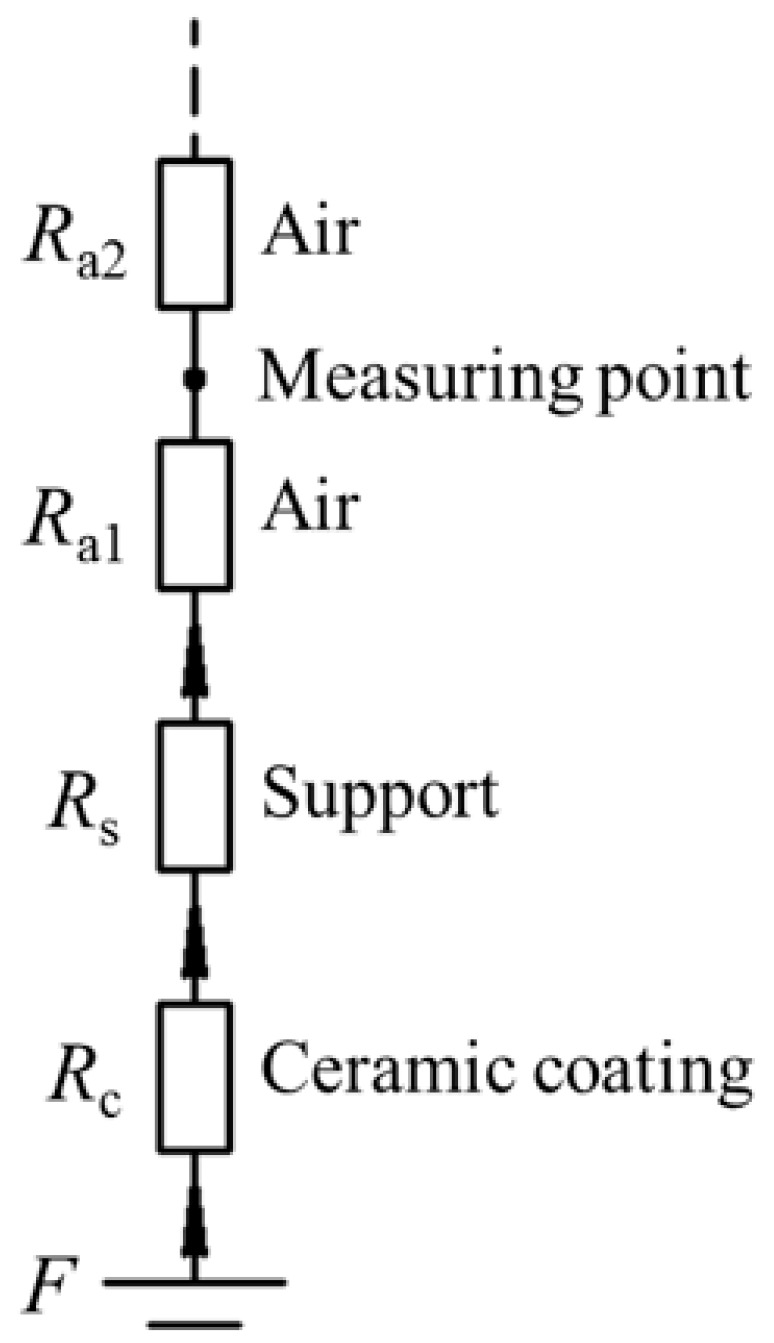
The model of the MFL sensing principle for steel pipe.

Based on Hopkinson’s law [50], the MFL sensing principle with the FMS is analyzed. As schematically displayed in Figure 3, from the pipe surface to the sensor measuring point there are ceramic, support, and air, and their magnetic reluctances are *R*_c_, *R*_s_, and *R*_a1_, respectively. From the measuring point to an infinite far place, the magnetic reluctance is *R*_a2_. Then, we can get:

(1) $$\phi =\frac{F}{R_{c}+R_{s}+R_{a1}+R_{a2}}$$

where *F* denotes the magnetomotive force depending on the defect size and magnetization intensity; *ϕ* denotes the leaked magnetic flux. The magnetic reluctance is expressed as follows:

(2) $$R=\frac{h}{\mu A}$$

where *h* is the lift-off distance, *μ* is the permeability of the material, and *A* is the cross-sectional area. Based on Equations (1) and (2), the leaked magnetic flux can be expressed as follows:

(3) $$\phi =\frac{F}{\frac{h_{c}}{\mu_{c}A}+\frac{h_{s}}{\mu_{s}A}+\frac{h_{a1}}{\mu_{a}A}+\frac{h_{a2}}{\mu_{a}A}}$$

where *μ*_c_, *μ*_s_, and *μ*_a_ denote the permeability of the ceramic coating, support, and air, respectively. *μ*_c_ is approximately equal to *μ*_a_. Finally, the relationship between the leaked magnetic flux *ϕ* and the support permeability *μ*_s_ can be obtained as follows:

(4) $$\phi =\frac{F}{\frac{h_{c}+h_{a1}+h_{a2}}{\mu_{a}A}+\frac{h_{s}}{\mu_{s}A}}$$

From Equation (4), the leaked magnetic flux *ϕ* and the support permeability *μ*_s_ has a positive correlation. When the non-ferromagnetic support is replaced by a high-permeability ferromagnetic support, the magnetic reluctance of the leaking path from the defect to the sensor location is reduced greatly, and thereafter more leaked flux leakage is guided to the sensor location, leading to a higher sensitivity. Besides, the proposed sensing method is simple and achievable, as it changes nothing to the conventional probe structure except the support material.

## 3. Numerical Simulation of the MFL Sensing Method Based on the Magnetic Guiding Effect

**Figure 4:**
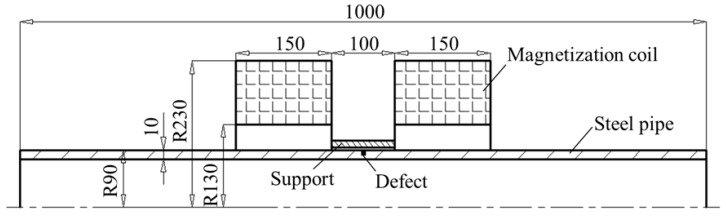
The simulation model of MFL testing for steel pipe.

In order to investigate the magnetic guiding effect of the FMS, numerical simulations are conducted by ANSOFT. It is well known that the MFL generated by the defects can be maximized only under the condition that the ferromagnetic object is fully magnetized to the saturation status. Thus, in the online MFL inspection of steel pipes, a Helmholtz coil magnetization method is proposed, which will produce a strong and uniform magnetizing field, as displayed in Figure 4. A steel pipe (material grade: J55; thickness: 10 mm; external radius: 90 mm) is analyzed, in which a circumferential defect (width: 1 mm; depth: 2.0 mm) is made. The parameters of the Helmholtz coil magnetizer are as follows: the internal radius of the coil is 130.0 mm, the external radius of the coil is 230 mm, the thickness of the coil is 150 mm, and the distance between the two coils is 100 mm. The thickness of the ceramic coating and support are 0.1 mm and 0.5 mm, respectively. Since the pipe and Helmholtz coil are axis-symmetric, finite element modeling and simulation procedures are implemented in two dimensions (2D). In mesh operation, the maximum length of elements is restricted to 0.05 mm.

**Figure 5:**
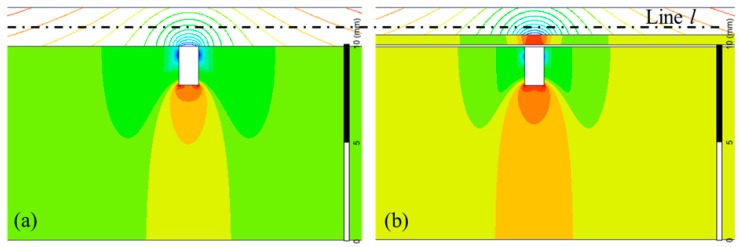
The MFL distribution. (**a**) The magnetic flux distribution with the NFMS; (**b**) the magnetic flux distribution affected by the FMS.

The MFL distributions with the NFMS (*μ*_s_ = *μ*_a_) and FMS (material: steel 1020) are simulated, as displayed in Figure 5a,b, respectively. With the same current density of 2.0 × 10^6^ A/m^2^ in the magnetization coils, the same defect generates different MFL distributions. In Figure 5a, the MFL generated by the defect is distributed freely, which is not influenced by the sensing device, because the ceramic coating and support are both non-ferromagnetic materials. Thus, the conventional sensing method is a passive method. However, with the FMS, more magnetic flux is guided in the FMS and then leak out into the air, as displayed in Figure 5b, which is an active sensing method.

**Figure 6:**
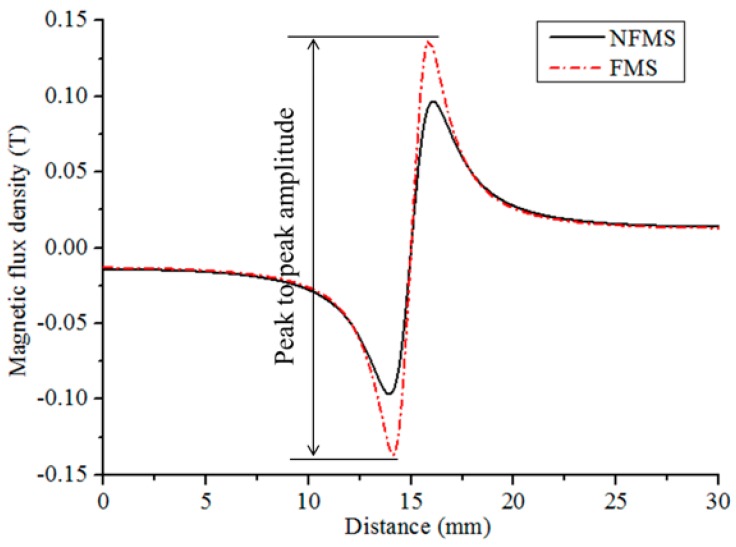
The MFL signals influenced by different supports.

Along the line *l* at the same lift-off distance of 1.0 mm from the pipe surface, as shown in Figure 5, the normal components of MFL are calculated and displayed in Figure 6. It can be seen that the MFL intensity with the FMS is obviously greater than that with the NFMS. Thus, the proposed method is valid to improve the MFL sensitivity. To accurately investigate MFL intensity, the peak-to-peak amplitude of the MFL signal is analyzed in the following part, as defined in Figure 6, which can eliminate the baseline drift [33].

**Figure 7:**
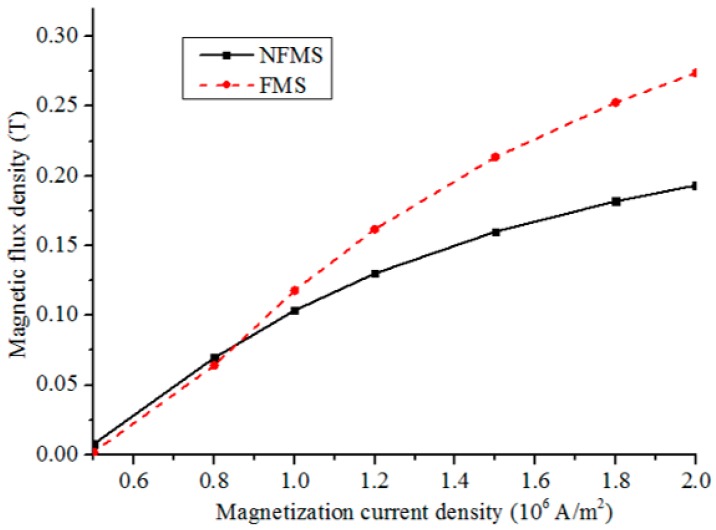
The MFL peak-to-peak amplitudes at different lift-off distances with different magnetization current densities.

To compare the conventional and proposed methods, the influence of the magnetization intensity on MFL intensity is simulated with a fixed lift-off distance of 1.0 mm. Figure 7 shows the peak-to-peak amplitudes of the normal component of MFL with different magnetization current densities from 0.5 × 10^6^ A/m^2^ to 2.0 × 10^6^ A/m^2^. It can be seen that at a low current density, the MFL intensity with the FMS is slightly smaller than that with the NFMS. With the FMS, more materials need to be magnetized and the nonlinear permeability of the FMS depends on the magnetization intensity. Thus, for the FMS, applying a weak magnetizing field will generate a small magnetomotive force *F* and a small FMS permeability *μ*_s_. Based on Equation (4), the MFL intensity with the FMS will be smaller than that with the NFMS. Then, with the magnetization current increasing to a higher level, the MFL intensity with the FMS is greater than that with the NFMS, and the difference becomes more and more obvious. Finally, at the saturation magnetization status, the difference becomes the largest, and the greatest MFL sensitivity is obtained. Thus, in the practical inspection, applying a strong magnetizing field is necessary for the proposed sensing method.

## 4. Experimental Study of the MFL Sensing Method Based on the Magnetic Guiding Effect

**Figure 8:**
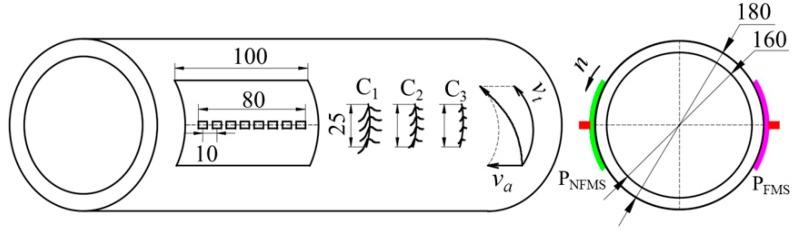
The online MFL probe for steel pipe (unit: mm).

**Figure 9:**
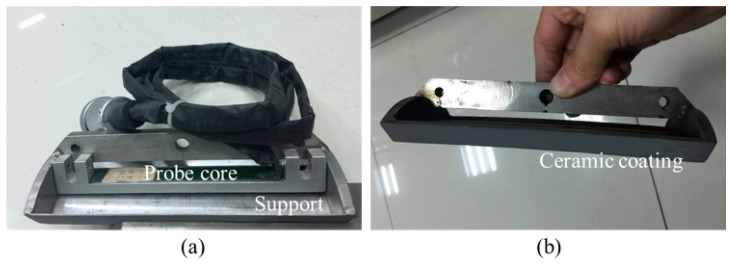
The online MFL probe for steel pipe. (**a**) The overall structure of the probe; (**b**) the ceramic coating painted on the support.

To validate the feasibility of the proposed method, MFL experiments are performed by using an online MFL testing system. A steel pipe (diameter = 180.0 mm; thickness = 10.0 mm; length = 8000.0 mm; material: J55) was inspected, as displayed in Figure 8. Three artificial circumferential defects are made in the pipe wall, i.e., defect C_1_ (depth = 1.0 mm; width = 0.5 mm; length = 25.0 mm), defect C_2_ (depth = 0.5 mm; width = 0.5 mm; length = 25.0 mm) and defect C_3_ (depth = 0.2 mm; width = 0.5 mm; length = 25.0 mm). In order to compare the sensitivity of the proposed method to the conventional method, a P_NFMS_ and a P_FMS_ are developed and tested, which have the same structure except the support material. The supports for P_NFMS_ and P_FMS_ are made of non-ferromagnetic stainless steel 302B and ferromagnetic steel 1020, respectively. Each probe has eight hall sensors, forming an effective length of 80 mm, and the sensor array is installed in a support with a length of 100 mm. As pictured in Figure 9a, the sensor array is packaged in a probe core, which is mechanically connected to the protecting support. The thickness of the ceramic coating is 0.1 mm and the thickness of the support is 0.5 mm. This way, only the support is consumable while the expensive probe core can be reused. As pictured in Figure 9b, the dark color denotes the ceramic coating. When the dark color of the support is grinded to a shiny color, the support needs to be replaced by a new one.

**Figure 10:**
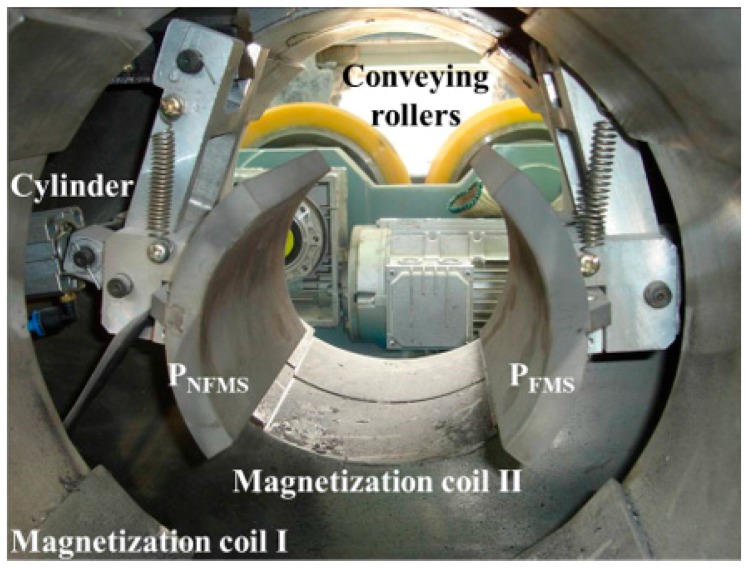
The MFL probing system for steel pipe.

**Figure 11:**
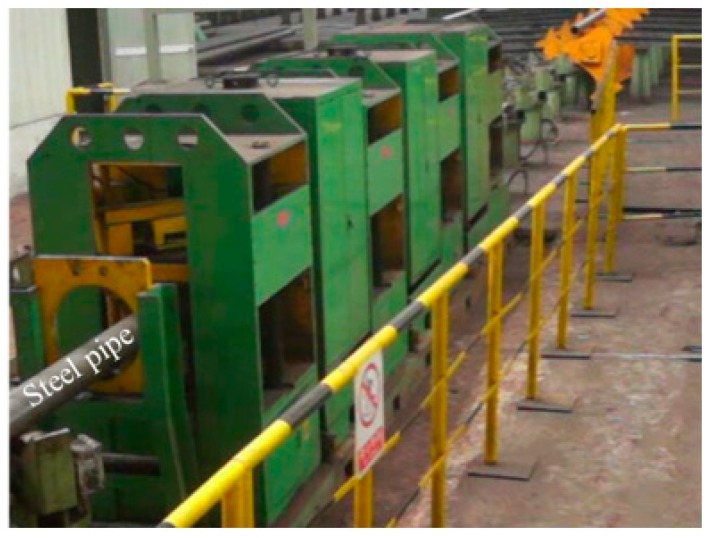
The whole online MFL inspection apparatus for steel pipe.

As pictured in Figure 10 and Figure 11, the steel pipe is driven forward in a helical motion with the axial pitch of 80 mm by speed-control conveying rollers. When the pipe arrives at the inspection apparatus, it is magnetized by the Helmholtz coils I and II, and then the two probes P_NFMS_ and P_FMS_ are forced to contact the pipe surface firmly by air cylinders to track the swing movement of the pipe. This way, all three defects can be scanned by both P_NFMS_ and P_FMS_.

**Figure 12:**
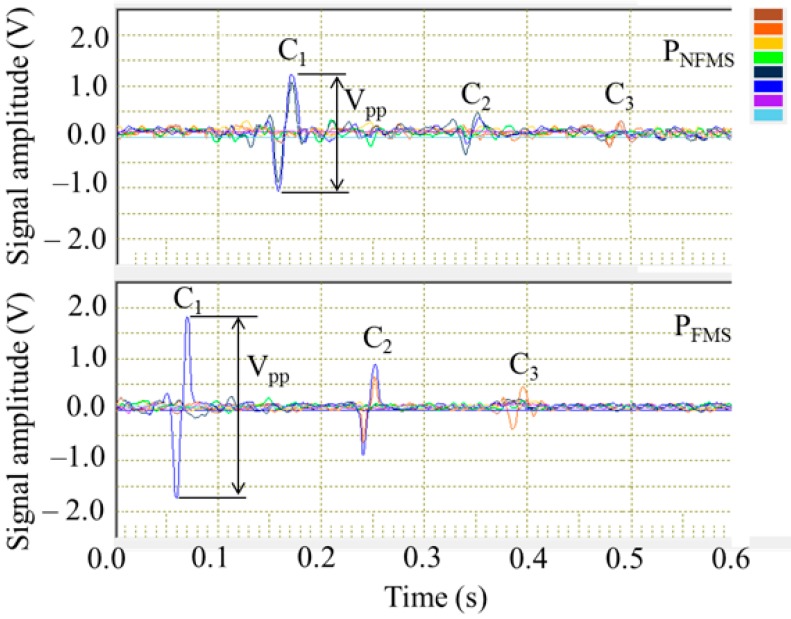
The testing signals captured by P_NFMS_ and P_FMS_.

With the magnetization current of 40 A in the Helmholtz coils, the testing MFL signals from the three defects are captured by P_NFMS_ and P_FMS_, respectively, as shown in Figure 12. The top and bottom parts display the signals from P_NFMS_ and P_FMS_, respectively. In each part, there are eight signal channels with eight different colors, displaying the eight hall sensors. The SNR of three defects scanned by P_NFMS_ and P_FMS_ are calculated and listed in Table 1. It can be seen that the proposed P_FMS_ has an obviously higher sensitivity than the conventional P_NFMS_, especially for the tiny defect C_3_. The defect C_3_ scanned by probe P_NFMS_ is nearly missing detection, while it generates a recognizable signal response by probe P_FMS_. The steel pipe is driven forward in a helical motion and the defects are first scanned by probe P_NFMS_ and then scanned by probe P_FMS_, thus, the signal response from P_FMS_ shows a time delay compared to that from P_NFMS_.

**Figure 13:**
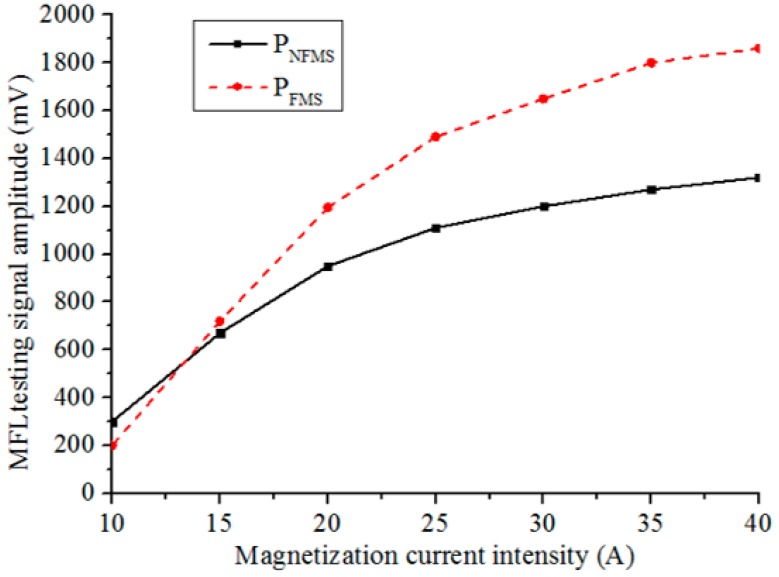
The testing amplitudes captured by P_NFMS_ and P_FMS_ with different currents.

Then, to investigate the influence of the magnetization status on the MFL sensitivity, different magnetization currents are applied to the Helmholtz coil from 10 A to 40 A. The peak-to-peak amplitude V_pp_ for the defect C_1_ is analyzed, as defined in Figure 12. The testing signal amplitudes scanned by P_NFMS_ and P_FMS_ with different magnetization currents are displayed in Figure 13, with the magnetization current intensity increasing, the signal amplitudes captured by P_NFMS_ and P_FMS_ are both increasing but with different rates, which match the simulation results well, as displayed in Figure 7. The peak-to-peak amplitude captured by P_FMS_ increases faster than that captured by P_NFMS_. When the magnetization current is increased to a high level, a great sensitivity difference is obtained. Thus, to obtain a high MFL sensitivity for steel pipe, saturation magnetization is necessary.

## 5. Conclusions

In order to improve the sensitivity of online MFL inspection for steel pipe, a MFL sensing method based on the magnetic guiding effect has been proposed in this paper. Different from the conventional passive sensing method, the proposed method utilizes a high-permeability FMS to guide more magnetic flux to leak out, which is an active sensing method. Simulations and experiments show that MFL intensity with the FMS increases faster than that with the NFMS during the increase of the magnetization current, leading to a higher sensitivity. Compared to the conventional contact method, the proposed method changes nothing to the probe structure except the support material, which is simple and achievable, which also can be used for other ferromagnetic material inspection, such as the online inspection for steel wire. Furthermore, to improve the MFL sensitivity for the proposed method, saturation magnetization is necessary.

## Figures and Tables

**Table 1:** The SNR comparison for P_NFMS_ and P_FMS_ (magnetization current = 40 A).

Probe	C_1_	C_2_	C_3_
P_NFMS_	16.5 dB	8.3 dB	/
P_FMS_	19.3 dB	13.4 dB	6.3 dB
